# Pds5A and Pds5B Display Non-redundant Functions in Mitosis and Their Loss Triggers Chk1 Activation

**DOI:** 10.3389/fcell.2020.00531

**Published:** 2020-07-14

**Authors:** Naif Al-Jomah, Lubinda Mukololo, Awais Anjum, Mohammed Al Madadha, Raj Patel

**Affiliations:** ^1^Department of Molecular and Cell Biology, University of Leicester, Leicester, United Kingdom; ^2^Molecular Oncology Department, Research Centre, King Faisal Specialist Hospital and Research Centre, Riyadh, Saudi Arabia; ^3^Department of Physiological Sciences, School of Medicine, University of Zambia, Lusaka, Zambia; ^4^School of Biosciences, University of Nottingham, Loughborough, United Kingdom; ^5^Department of Pathology, Microbiology and Forensic Medicine, School of Medicine, The University of Jordan, Amman, Jordan

**Keywords:** Pds5A, Pds5B, cohesin, mitosis, DNA damage, ATR, Chk1, spindle assembly checkpoints

## Abstract

**Background:**

Pds5 is an abundant HEAT-repeat-containing protein that binds to cohesin and mediates sister chromatid cohesion. In vertebrates, Pds5A and Pds5B are known to protect DNA replication fork, as their loss leads to DNA damage. Pds5 interacts directly with Wapl, to remove cohesin during mitosis.

**Aim:**

To analyze the effects of the loss of Pds5 proteins-mediated DNA damage on the cell cycle checkpoints and to examine the possibility that Pds5 proteins have an overlapping function.

**Methods:**

We first analyzed the cell cycle regulation of Pds5 proteins and defects in S-phase; DNA damage was confirmed after Pds5A/B knockdown. The activation of cell cycle checkpoints and apoptosis were examined by the level of p-Chk1^S317^, MAD2 localization, and the level of pro-apoptotic markers, respectively.

**Results:**

Pds5 proteins dissociated from chromatin in a stepwise manner, and their loss led to activation of pro-apoptotic markers associated with the phosphorylation of Chk1^S317^ due to DNA damage. Depletion of either Pds5A or Pds5B alone increased Smc3 acetylation in perturbed cell cycle, while depletion of both proteins severely impaired Smc3 acetylation. Moreover, the loss of Pds5A/Pds5B activated the SAC in an ATR-Chk1-dependent manner and stabilized Wapl on chromatin. The depletion of Chk1 rescued the S-phase delay associated with Pds5 depletion and significantly increased mitotic catastrophe.

**Conclusion:**

Pds5A and Pds5B display overlapping functions in facilitating Smc3 acetylation. Somewhat paradoxically, they also have non-redundant functions in terms of cohesin removal due to the activated surveillance mechanism that leads to phosphorylation of Chk1^S317^.

## Introduction

Sister chromatid cohesion is mediated by the multi-subunit cohesin complex, which comprises four core proteins Smc1, Smc3, kleisin subunit Scc1 (Rad21 in humans), and Scc3 (SA1 or SA2 in vertebrates), and the regulatory proteins include sororin, wings apart-like (Wapl), and Pds5 (Schmitz et al., 2007; Shintomi and Hirano, 2009; Nasmyth, 2011; Losada, 2014). The establishment and maintenance of sister chromatid cohesion are aided by the replisome component CHl1 helicase and Smc3 acetylation by the replication fork-associated acetyltransferase Eco1/Ctf7 (Esco1 and Esco2 in humans) (Skibbens et al., 1999; Toth et al., 1999; Ivanov et al., 2002; Unal et al., 2007; Nishiyama et al., 2010; Samora et al., 2016). Wapl associates with cohesin by interacting with a specific amino acid sequence on cohesin’s Scc1 and SA1/SA2 subunits via the FGF motif present in the N-terminus of Wapl (Kueng et al., 2006; Shintomi and Hirano, 2009). Sororin and Wapl have opposite functions in regulating sister chromatid cohesion. Sororin competes with Wapl to bind Pds5 and antagonize Wapl to maintain sister chromatid cohesion during interphase (Nishiyama et al., 2010).

In higher eukaryotes, the dissociation of cohesin from chromosomes during mitosis is highly regulated. Phosphorylation of sororin by Cdk1 inhibits its binding to Pds5 (Nishiyama et al., 2013), allowing the binding of Wapl to Pds5 and hence the removal of cohesin from the chromosome arms. However, centromeric cohesin is protected from degradation by shugoshin 1 (Sgo1). Sgo1 recruits a serine/threonine phosphatase, 2A (PP2A), which suppresses the phosphorylation of centromeric cohesin during prophase (Kitajima et al., 2006; Riedel et al., 2006; Tang J. et al., 2006). Following bipolar attachment of sister kinetochores to spindle microtubules, the anaphase-promoting complex (APC)-dependent activation of a peptidase (Separase) results in the degradation of residual centromeric cohesin to initiate anaphase (Hauf et al., 2001). Cdc20, a substrate-specific activator of APC/C, is the target of spindle assembly checkpoint (SAC) activation (Yu, 2002).

The response to DNA damage and the activation of SAC cooperate and function together as a genome surveillance mechanism to avoid genomic instability and to ensure cell division fidelity (Losada, 2014; Tsutsumi et al., 2014; Zeman and Cimprich, 2014). In response to DNA damage, sensor proteins associate with the lesion to recruit transducers of the damage signal and trigger the DNA damage checkpoint response by activating the signal transducer kinase ATM/ATR, which in turn activates the checkpoint kinases Chk1 and Chk2 (Liu et al., 2000; Matsuoka et al., 2000; Heyer et al., 2010). In metazoans, a well-characterized function of Chk1 activation in response to DNA damage or replication inhibitors is to maintain the stability of replication forks, inhibit the firing of origins, and delay entry of the cell into mitosis (Nurse, 1997; Feijoo et al., 2001). Chk1 can also phosphorylate mitotic arrest deficient protein Mad2 *in vitro*, and Chk1 loss leads to Mad2 down regulation (Chila et al., 2013). Mad2 and the histone variant CENPA become enriched at the nuclear periphery in a manner dependent on the response to DNA damage (Foley and Kapoor, 2013).

The Precocious Dissociation of Sister protein (Pds5) is a member of a highly conserved family of proteins which was initially identified as an essential factor for the establishment and maintenance of sister chromatid cohesin during S-phase (Hartman et al., 2000). In mammalian cells, there are two variants of Pds5, Pds5A (1337 amino acids) and Pds5B/APRIN (1447 amino acids), and both interact with cohesin and regulate its removal from chromatin (Losada et al., 2005; Gause et al., 2010). Several lines of evidence indicate the role of Pds5B in DNA damage repair and homologous recombination (HR) (Brough et al., 2012; Kusch, 2015; Couturier et al., 2016). Notably, the direct interaction between Pds5B and BRCA2, a protein associated with DNA repair, has been shown to regulate HR and prevent replication fork stalling (Boulton, 2006; Schlacher et al., 2011). Moreover, low Pds5B expression levels predict better survival in patients with breast and ovarian cancer, as the loss of Pds5B sensitizes breast cancer cells to DNA-damaging chemotherapy (Brough et al., 2012). It has recently been shown that both Pds5A and Pds5B are essential for replication fork protection. They recruit WRN helicase-interacting protein 1, RAD51 recombinase, and BRCA2 DNA repair associated with stalled forks (Morales et al., 2020), and their loss induces DNA double-strand breaks. Nevertheless, this does not affect the cellular levels of phosphorylated Chk1 (S345) (Carvajal-Maldonado et al., 2019).

In the current study, we sought to analyze the impact of Pds5 loss-of-function on the surveillance mechanism and to determine the upstream SAC regulator that blocks cell entry into anaphase. We report that the depletion of Pds5A or Pds5B or both induced phosphorylation of Chk1 (S317) with concomitant Smc3 acetylation and DNA damage-mediated stalling of DNA replication forks in perturbed and unperturbed cell cycle. This also led to SAC activation in an ATR-Chk1-dependent manner. Surprisingly, the Pds5 depletion-induced inhibition of DNA replication and SAC activation were rescued by Chk1 depletion, and they were accompanied by a significant increase in cell death mediated by mitotic catastrophe.

## Results

### Pds5A and Pds5B Dissociate From Chromatin in a Stepwise Manner

**FIGURE 1:**
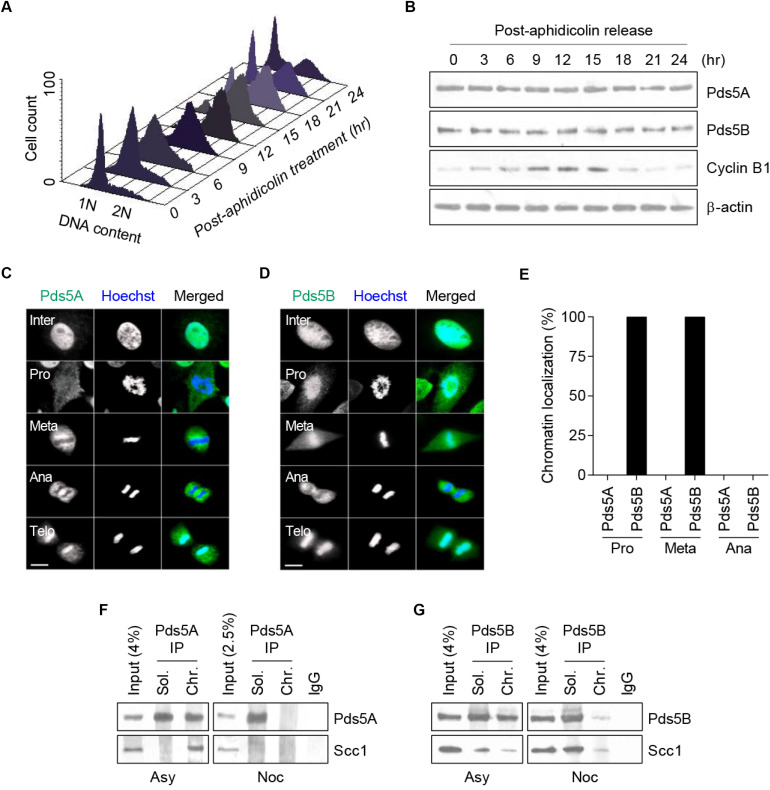
Analysis of endogenous Pds5A and Pds5B expression and intracellular distribution during HeLa cell cycle. **(A)** HeLa cells were synchronized in the G1/S phase by aphidicolin block and release and were analyzed by FACS at a series of time points. **(B)** Total protein extracts were prepared at the indicated time points and analyzed by western blot against Pds5A, Pds5B, cyclin B1, and actin. **(C,D)** Immunofluorescence microscopy images showing the intracellular distribution of Pds5A and Pds5B (green) at different stages of the cell cycle. DNA was stained with Hoechst 33342 (blue). Merged images are shown (right panel). Scale bar: 8 μm. This figure is representative of three independent experiments. **(E)** Histogram showing the percentage of cells showing the differential disassociation of Pds5A and Pds5B during mitosis. **(F,G)** Soluble and chromatin fractions from asynchronous and nocodazole-arrested HeLa cells were subjected to Pds5A or Pds5B immunoprecipitation and were analyzed by western blot against Pds5A, Pds5B, and Scc1.

First, we sought to determine the expression levels of the Pds5A and Pds5B proteins and their intracellular localization at specific stages of the cell cycle in a synchronized population of human cervical carcinoma cells (HeLa). This was achieved by exposing cells to G1/S block and release using aphidicolin, an inhibitor of DNA polymerase α (Krokan et al., 1981). FACS analysis revealed that mitosis occurred 15 h after release from the aphidicolin block (Figure 1A). Data obtained from the time-course experiment showed that Pds5A and Pds5B protein expression levels remained constant throughout the cell cycle (Figure 1B). Cyclin B1 was used as a marker for mitosis. Immunofluorescence data revealed that during the interphase, Pds5 proteins predominantly localized to the nucleus and only dissociated from chromatin at mitosis. Surprisingly, Pds5A and Pds5B dissociated from chromatin in a stepwise manner, at prophase and at the anaphase-metaphase transition, respectively (Figures 1C–E). Both Pds5A and Pds5B re-associated with chromatin at the telophase. The delayed dissociation of Pds5B from chromatin was further confirmed by analysis of the Pds5A and Pds5B immunoprecipitates of chromatin-associated proteins derived from asynchronous and nocodazole-arrested cells (Figures 1F,G). The cohesin subunit, Scc1, was found to co-immunoprecipitate only with Pds5B, not with Pds5A in nocodazole-arrested cells. The disparity observed in the chromatin residence time of Pds5 proteins suggests that these proteins could regulate sister chromatid cohesion differently and might have non-overlapping functions.

### Pds5A and Pds5B Display Redundant Functions in the S-Phase but Not in Mitosis

**FIGURE 2:**
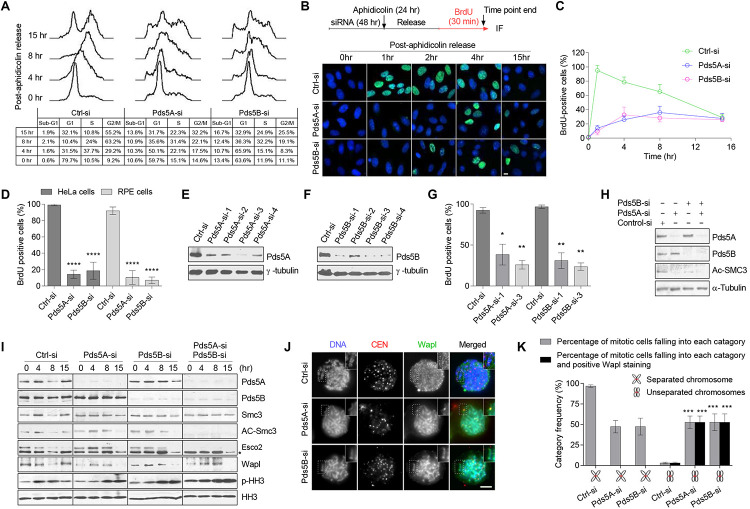
Pds5A and Pds5B have overlapping functions in S-phase but distinct roles in mitosis. **(A)** FACS analysis of Pds5-depleted HeLa cells at the indicated time points following release from G1/S-phase arrest. **(B)** The experimental scheme is shown (top). Immunofluorescence images of Pds5-depleted HeLa cells labeled with BrdU for 30 min before the end of the indicated time point after release from G1/S-phase arrest. DNA was stained with Hoechst 33342 (blue). Scale bar: 10 μm. **(C)** Graph showing quantitation of the data shown in **(B)**. At least 100 cells were counted in randomly selected fields. Each point represents the mean ± SEM of three independent experiments. **(D)** Histogram showing the percentage of BrdU-positive cells. Exponentially growing HeLa and RPE cells were labeled with BrdU for 30 min after 48 h from transfection with 50 nM control-si, Pds5A-si, or Pds5B-si. At least 100 cells were counted in randomly selected fields. Data represent the mean ± SEM of three independent experiments: ****p < 0.0001. *P*-values were calculated using two-way ANOVA. **(E,F)** HeLa cells were separately transfected with individual siRNAs directed against Pds5A or Pds5B for 48 h. Total cell extracts were prepared and subjected to immunoblotting analysis using antibodies against the indicated proteins. **(G)** Histogram showing the percentage of BrdU-positive cells following individual siRNAs treatment. Mean values ± SD of measurements are from at least 100 cells from three independent experiments: *p < 0.05; **p < 0.01. *P*-values were calculated using two-way ANOVA. **(H)** Reduction of Ac-Smc3 was determined by immunoblotting analysis of total cell lysates prepared from HeLa cells after depletion of Pds5 proteins. **(I)** Immunoblotting analysis of chromatin-associated protein fractions was prepared from synchronized HeLa cells at the G1/S-phase with aphidicolin for 24 h after depletion of Pds5A, Pds5B, and both. Phospho-histone H3 (PHH3) was used as a marker for mitosis. Asterisk indicates a nonspecific band. **(J)** Representative immunofluorescence microscopy images showing the distribution of Wapl in HeLa cells arrested at mitosis with 10 μM Taxol after depletion of Pds5A or Pds5B by siRNA; enlarged images are shown in the insets. Scale bar: 8 μm. **(K)** Histogram indicating the frequency of Wapl localization along unresolved chromosome arms after depletion of Pds5A or Pds5B. Mean values ± SD of measurements from at least 100 cells from three independent experiments: ***p < 0.001. *P* values were calculated using two-way ANOVA.

To examine the role of Pds5 proteins in the S-phase, HeLa cells with a low passage number were transfected with a pool of four small, interfering RNAs (siRNAs) to specifically deplete the Pds5A and Pds5B proteins. We observed an effective depletion of Pds5A and Pds5B (Supplementary Figures S1A–E), while the other cohesin regulatory proteins were not affected (Supplementary Figure S1F). Next, we treated HeLa cells with control-si, Pds5A-si or Pds5B-si for 48 h before synchronizing them at the G1/S boundary using aphidicolin for 24 h. The cell cycle progression through S-phase, following the removal of aphidicolin, was monitored using flow cytometry (Figure 2A). The Pds5-depleted cells showed a remarkable slow cell cycle progression and increase in the sub-G1 peak, indicating the presence of apoptotic cells.

BrdU-labeling of a synchronized population of Pds5-depleted cells, following aphidicolin block and release, further confirmed the delay in DNA replication (Figures 2B,C). Analysis of DNA replication in an asynchronous population of either Pds5-depleted HeLa cells or Pds5-depleted non-transformed retinal pigment epithelial cells (Bodnar et al., 1998) revealed a significant reduction in BrdU incorporation in comparison with the control-si-treated cells (Figure 2D). To eliminate the possibility that we were observing the off-target effect, we used individual siRNAs directed against Pds5A or Pds5B (Figures 2E,F). Since different Pds5 siRNAs can inhibit the DNA replication in HeLa cells (Figure 2G), it is unlikely that this is an off-target effect of the siRNAs.

Next, we sought to monitor the state of Smc3 acetylation after the depletion of Pds5 proteins. In asynchronous Pds5-depleted cells, the level of Smc3 acetylation was remarkably reduced (Figure 2H). These results are consistent with a previous study suggesting that Pds5 proteins are required to maintain Smc3 acetylation (Carretero et al., 2013). We then sought to analyze the state of Smc3 acetylation in synchronized Pds5-depleted cells. We depleted Pds5A and Pds5B, individually or simultaneously, from HeLa cells before synchronization at the G1/S phase boundary using aphidicolin. Chromatin fractions were prepared and analyzed by immunoblotting. Figure 2I shows that the depletion of Pds5A or Pds5B increased the level of Smc3 acetylation, which remained high even after release from the aphidicolin block as compared with control-si-treated cells. However, depletion of both Pds5 proteins abolished Smc3 acetylation, and was associated with substantial decrease in the chromatin-associated Esco2 (Figure 2I), which would, in turn, suggest that Pds5 variants have redundant functions.

Importantly, Wapl recruitment to chromatin was affected by the depletion of Pds5 proteins, either individually or in combination, notably at 0 h (Figure 2I), whereas, the dissociation of Wapl from chromatin was delayed from 8 h in control-si to 15 h in Pds5-depleted cells. To confirm this effect in mitosis, we transfected cells with control-si, Pds5A-si, or Pds5B-si for 48 h prior to synchronization at mitosis with Taxol for 24 h, and then examined the intracellular localization of Wapl using immunofluorescence microscopy. We found an increased number of unresolved sister chromatids in cells depleted of Pds5A or Pds5B, and Wapl was found to be present along the entire length of all unresolved sister chromatids (Figures 2J,K), consistent with the notion that the cohesin removal function of Pds5 cannot be compensated for by the depletion of either Pds5 variant.

### Loss of Pds5 Leads to Activation of ATR/Chk1 Signaling

**FIGURE 3:**
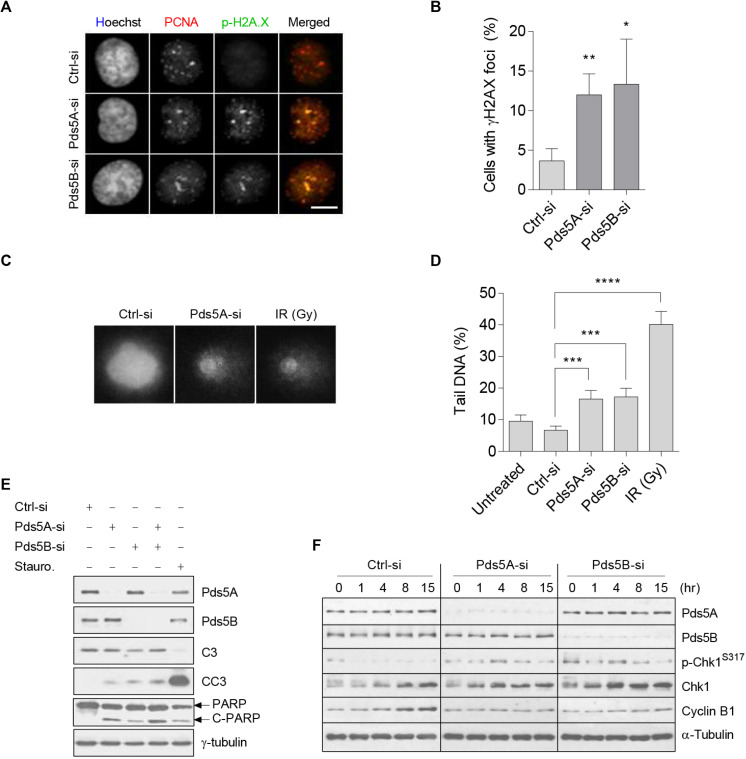
Loss of Pds5A and Pds5B-induced DNA damage, Chk1 phosphorylation, and apoptosis. **(A)** Immunofluorescence images of Pds5-depleted cells showing the phosphorylation and foci formation of histone H2AX (green) that co-localizes with PCNA (red). DNA was stained with Hoechst 33342 (blue). Merged images are shown on the right. Scale bar: 8 μm. **(B)** Histogram indicating the percentage of cells with phospho-histone H2AX foci, with the mean ± SEM of at least 100 cells from three independent experiments shown: **p < 0.01; *p < 0.05. *P-*values were calculated using a two-tailed Students *t-*test. **(C)** Comet images of propidium iodide (1 mg/ml)-labeled HeLa cells following a 48 h treatment with 50 nM of either control-si or Pds5A-si. Similar results were obtained with cells depleted of the Pds5B protein. Cells were mixed with low melting point agarose prior to electrophoresis in ice-cold alkali buffer at 30 V, 300 mA for 20 min. Control cells were also X-ray-irradiated (10 Gy). Scale bar: 10 μm. **(D)** Histogram showing quantitation of data in **(C)**. The DNA damage is expressed as the percentage of DNA in the comet tails. The mean ± SEM of at least 100 cells scored in randomly selected fields from three independent experiments are shown: **p < 0.01; ***p < 0.001; ****p < 0.0001. *P* values were calculated using a two-tailed Student’s *t*-test. **(E)** Western blot analysis of Pds5-depleted cell lysates with antibodies against Pds5A, Pds5B, caspase-3, cleaved caspase-3, PARP, cleaved PARP, and γ-tubulin. Parallel cells were treated with 1 μM of staurosporine for 6 h to induce apoptosis. **(F)** Western blot detection of p-Chk1^S317^ in response to DNA stress. Total protein extracts were prepared from synchronized control cells and Pds5A- or Pds5B-depleted cells at the G1/S phase and released for the indicated time points.

Previous studies suggested that the depletion of Pds5 leads to DNA double-strand break (Carvajal-Maldonado et al., 2019). In this regard, we speculated that this damage might activate a surveillance mechanism that operates upstream of the SAC. Therefore, Co-immunofluorescence staining of phospho-histone H2AX and PCNA was performed following the depletion of Pds5A/B. In cells depleted of Pds5A/B, the phosphorylated histone H2AX co-localized with PCNA (Figures 3A,B), indicating the presence of DNA double-strand breaks and the stalling of the replication fork. As an additional readout of DNA damage, we performed a comet assay (Figures 3C,D), and found a significant increase in the proportion of tail DNA in Pds5-depleted cells in comparison with the control, which in turn triggered apoptosis, as evidenced by both cleaved Parp and Caspase 3 (Figure 3E). Given that DNA damage or replication stress leads to the activation of ATR/ATM protein kinases (Kurose et al., 2006a, b), we sought to investigate whether it also affects the cellular levels of p-Chk1. To this purpose, we found that Chk1 remained phosphorylated in the Pds5-depleted cells at all-time points analyzed by immunoblotting (Figure 3F), suggesting the activation of the intra-S-phase checkpoint.

### The SAC Contributes to the DNA Damage Response Caused by the Loss of Pds5

**FIGURE 4:**
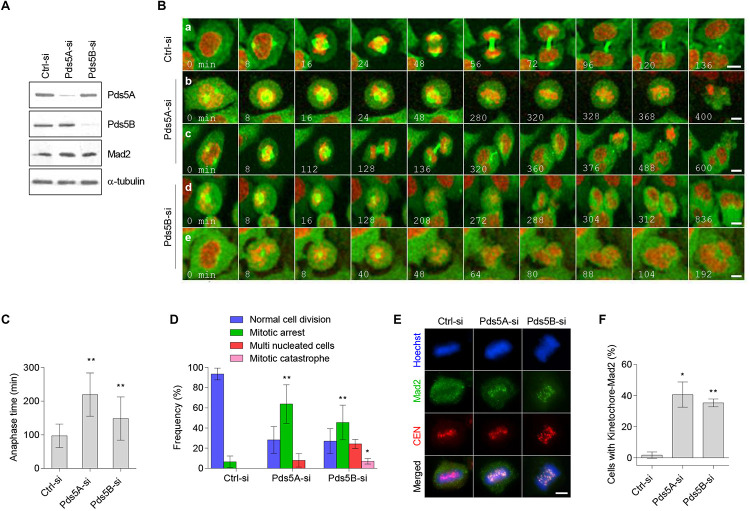
Time-lapse confocal microscopy of Pds5A- and Pds5B-depleted cells. HeLa cells stably expressing mCherry-histone H2B and α-tubulin-EGFP were transfected with the control, Pds5A-si, or Pds5B-si. After 48 h, cells were subjected to immunoblotting analysis with the indicated antibodies **(A)**, or they were analyzed by time lapse recording and imaged every 8 min to monitor their progression through mitosis **(B)**. **(B)** Selected images depicted from prophase; time is shown in min: (a) control-si; (b) prolonged metaphase arrest; (c,d) defective anaphase and generation of cells with multiple nuclei; (e) mitotic catastrophe. **(C)** Histogram representing the anaphase time in Pds5A- and Pds5B-depleted cells in comparison with the control. Mean values ± SD are shown: **p < 0.01. *P-*values were calculated using one-way ANOVA. **(D)** Quantification of the cell cycle defects seen in **(B)**. Mean values ± SD of counts from four independent experiments are shown: **p < 0.01; *p < 0.05. *P*-values were calculated using two-way ANOVA. **(E)** Immunofluorescence microscopy images showing the co-localization of Mad2 with kinetochore at the metaphase in HeLa cells depleted of Pds5A and Pds5B. Scale bar: 8 μm. **(F)** Histogram showing the percentage of the cells indicating co-localization of Mad2 with kinetochore at metaphase in HeLa cells depleted of Pds5A and Pds5B, with mean values ± SD from three independent experiments shown: **p < 0.01; *p < 0.05. *P-*values were calculated using the Student’s *t*-test.

We next sought to determine the effect of Pds5 protein depletion on mitotic progression using time-lapse fluorescence microscopy. HeLa cells stably expressing both mCherry-histone H2B and EGFP-α-tubulin were transfected with either Pds5A-si or Pds5B-si for 48 h. The depletion of the Pds5 proteins was confirmed by immunoblotting (Figure 4A) before imaging. For the time-lapse fluorescence microscopy experiments, we determined the time required by cells to progress from the prophase (the onset of chromosome condensation) to anaphase A (the onset of poleward movement of the chromosomes). Live-cell imaging of the control-si-treated cells indicated that anaphase A occurred at 97 ± 34.9 min (Figures 4B,C and Supplementary Movie 1), and that 88% of the cells analyzed completed normal chromosome segregation and cytokinesis (Figure 4D). In contrast, 64% of Pds5A-depleted cells and 45% of Pds5B-depleted cells experienced a significant delay in the onset of anaphase A (Figures 4A,D). Pds5A-depleted cells required 219 ± 64.25 min, and Pds5B-depleted cells required 148.1 ± 64.27 min to initiate anaphase A (Figure 4C and Supplementary Movies 2, 3).

Crucially, two significant outcomes were observed in the Pds5-depleted cells following prolonged mitosis, including apoptosis following a prolonged metaphase (Figure 4Bb and Supplementary Movie 4) and abnormal mitosis. Defective metaphase-anaphase transition frequently resulted in the generation of cells with micronuclei (Figures 4Bc,d,D, and Supplementary Movies 5, 6) or mitotic catastrophe (Figures 4B,e,D and Supplementary Movie 7). We next examined the localization of mitotic checkpoint protein Mad2 in Pds5-depleted cells using immunofluorescence microscopy. Figures 4E,F show that in Pds5A- or Pds5B-depleted cells, Mad2 co-localized with the kinetochores in metaphase cells, suggesting the activation of the SAC.

### Depletion of Chk1 Rescues the S-Phase Inhibition Caused by Pds5 Depletion Followed by Mitotic Slippage

**FIGURE 5:**
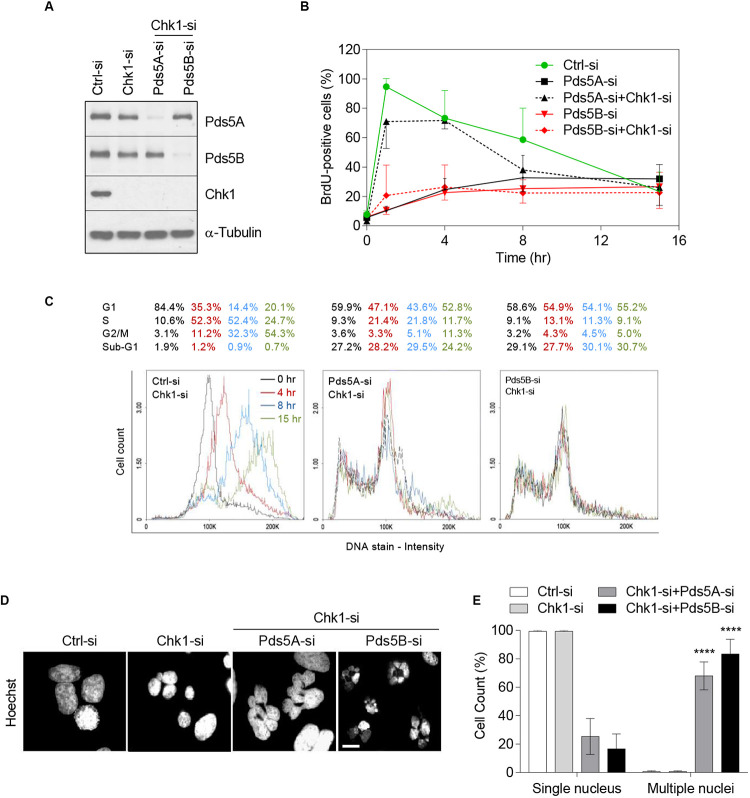
Chk1 depletion rescues the delay in DNA replication caused by the loss of Pds5 proteins and increases mitotic catastrophe. HeLa cells were treated with siRNA targeting Chk1 alone or both Chk1 and Pds5A or Pds5B, and were either subjected to immunoblotting analysis for Pds5A, Pds5B, and Chk1 **(A)**, or were synchronized at the G1/S phase and then collected at the indicated time points for BrdU quantification **(B)**. **(C)** FACS analysis to monitor S-phase progression in synchronized HeLa cells depleted of Chk1 alone or both Chk1 and Pds5A or Pds5B. **(D)** Immunofluorescence staining of cells undergoing mitotic catastrophe after co-transfection with Chk1 and Pds5A-si or Pds5B-si. Scale bar: 10 μm. **(E)** Histogram showing the percentages of mitotic catastrophe seen in **(D).** Mean values ± SD of the measurements from at least 100 cells from three independent experiments are shown: ****p < 0.0001. *P*-values were calculated using two-way ANOVA.

Next, we efficiently co-depleted Chk1 and either Pds5A or Pds5B in HeLa cells (Figure 5A). Whole-cell BrdU-labeling experiments in synchronized HeLa cells indicated that depletion of Pds5A alone resulted in the inhibition of the S-phase. As shown in Figure 5B, 95 and 10.3%, respectively, of the control and Pds5A-depleted cells incorporated BrdU 1 h after release from the aphidicolin block. However, the co-depletion of both Pds5A and Chk1 caused a dramatic rescue of the incorporating BrdU (from 10.3 to 71% of cells incorporating BrdU in 1 h). However, this rescue was minor in cells depleted of both Pds5B and Chk1, with only 20% of cells staining positively for BrdU at 1 h post aphidicolin release. The rescue of DNA replication in the absence of Pds5 and Chk1 was confirmed by flow cytometric analysis of the cells at intervals after release from the aphidicolin block (Figure 5C). Further analysis of cells depleted of Pds5A and Chk1 by flow cytrometry showed a noticeable rescue in S-phase progression, but not when both Pds5B and Chk1 were depleted (Figure 5C). In addition, the co-depletion of Chk1 and Pds5 proteins caused remarkable sub-G1 accumulations, suggesting the induction of apoptotic cell death. Qualitative and quantitative analysis of the asynchronous Chk1- and Pds5-depleted cells by fluorescence microscopy displayed a significant increase in the number of surviving cells with abnormal nuclei (Figures 5D,E), a common feature of mitotic catastrophe (Mc Gee, 2015). These results demonstrate that loss of Pds5, from cells with abrogated or compromised G2 checkpoint, the last opportunity to halt the cycle and repair DNA damage, lead to catastrophic mitosis followed by mitotic slippage prior to cell death.

## Discussion

In the current study, we uncovered the impact of the Pds5 loss-of-function on the surveillance mechanism. In particular, we identified the checkpoint response to DNA damage caused by depletion of Pds5A and Pds5B. We found that Pds5A and Pds5B have similar patterns in their expression levels but they differ in the timing of their dissociation. This is consistent with the Pds5 function in centromeric cohesion reported previously (Carretero et al., 2013). Some aspects of the function of Pds5A and Pds5B proteins in cell cycle regulation, namely the surveillance mechanism, remain unclear (Carvajal-Maldonado et al., 2019). Moreover, the difference in the function of Pds5A and Pds5B is poorly understood.

Previous studies on budding yeast have shown that depletion of Pds5 does not affect either the stability of chromatin-associated cohesin or its distribution along the chromosome (Kulemzina et al., 2012). The removal of cohesin at mitosis is highly regulated, and Pds5 plays an essential role in that process (Losada et al., 2005). Pds5 forms a complex with Wapl, and together they unload cohesin from sister chromatids (Nishiyama et al., 2010). Taking an RNAi approach, we found that the depletion of Pds5A and Pds5B individually or together caused defects in the S-phase with pronounced slow DNA replication in both HeLa cells and RPE cells. A previous study in MEFs showed reduced Smc3 acetylation and Wapl recruitment to chromatin after the loss of Pds5 proteins. This was attributed to a larger fraction of G1 cells and a smaller fraction of cells in S phase (Carretero et al., 2013). Consistent with this, we showed that loss of Pds5 proteins reduced Smc3 acetylation in unperturbed cell cycle. However, contrary to this observation, Smc3 acetylation was increased in the perturbed cell cycle of Hela cells-depleted of either Pds5A or Pds5B, but not both. Since Smc3 acetylation occurs specifically during S phase, synchronized cell cycle progression could explain, at least in part, the increase of Smc3 acetylation. Furthermore, Wapl recruitment to chromatin was elevated after release from G1/S block, and its dissociation was delayed and caused mitotic defects. In this regard, it is possible to speculate that Smc3 acetylation opposed the antiestablishment activity of Wapl (Rowland et al., 2009; Feytout et al., 2011). Analysis of mitotic cells revealed a great fraction of cells in which Wapl persisted along the unresolved chromosomes. Based on these observations, we concluded that Pds5A and Pds5B have an overlapping function in their contribution to cohesin acetylation, and that the two proteins might regulate different pools of cohesin on chromatin, with Pds5B being restricted to centromeric cohesin. But they cannot compensate each other for cohesin removal and that Wapl-independent releasing activity was switched off as cells activate SAC.

An earlier study (Uhlmann and Nasmyth, 1998) showed that cohesin Smc3 acetylation is needed to maintain cohesion establishment during DNA replication, while a recent study on *Drosophila* found that Pds5 was required to facilitate SA cohesin subunit binding near the origin of DNA replication (Misulovin et al., 2018). Consistent with this, Depletion of Pds5 proteins significantly increased the number of stalled replication forks and decreased fork velocity, notably in perturbed cell cycle (Carvajal-Maldonado et al., 2019). We further demonstrated that the loss of Pds5 proteins led to increased phosphorylated histone H2AX foci with PCNA, reduced cell survival, and induced apoptosis. This indicated the presence of DNA double-strand breaks that were induced by the loss of Pds5 proteins. Based on these results, we propose that the delay in the S-phase and the increased Smc3 acetylation observed in synchronized Pds5-depleted cells is a response of the intra-S-phase DNA damage checkpoint. Therefore, we set out to determine the critical effector of the surveillance mechanism that may operate upstream of the SAC upon Pds5A and Pds5B loss-of-function and that may also indirectly control Wapl-independent cohesin releasing activity.

Recent studies have shown that the loss of Pds5A or Pds5B has no influence on p-Chk1 (S345) (Carvajal-Maldonado et al., 2019). However, we found that the depletion of Pds5A or Pds5B or both led to a marked activation of the DNA damage checkpoint and increased the cellular level of phosphor-Chk1 at S317. Chk1 is known to play a major role in the signal transduction pathway (Nahse et al., 2017), and it is involved in the regulation of the SAC (Zachos et al., 2007). Accordingly, using cell imaging and immunofluorescence staining, we found a significant increase in prolonged anaphase time and mitotic arrest in addition to almost 40% more Mad2-positive kinetochores associated with the loss of Pds5A or Pds5B, which further indicates the activation of the SAC. Recent work has attributed the activation of Chk1 to the DNA damage caused by the depletion of Pds5 proteins; hence, the delay in the S-phase progression, the increased Smc3 acetylation, and the antiestablishment activity of Wapl are under the control of ATR-Chk1.

To confirm the requirement of Chk1 for SAC-dependent metaphase arrest in Pds5-depleted cells, we co-depleted Pds5 proteins with Chk1. As a result, the number of cells displaying mitotic catastrophes was increased up to fivefold, suggesting that severe DNA damage was induced by the depletion of the Pds5 proteins. Moreover, we showed that the inhibition of DNA replication due to Pds5 depletion was rescued by Chk1 depletion. Curiously, and according to this observation, the inhibition of DNA replication was more readily rescued when both Pds5A and Chk1 were depleted. This suggests different functional requirements for Pds5A and Pds5B. In particular, it has been suggested that Pds5B is essential for BRCA2 and Rad51 recruitment at damaged sites during HR-mediated repair (Brough et al., 2012; Kusch, 2015; Couturier et al., 2016). More noteworthy is that when Pds5A-depleted cells were viewed by live-cell imaging, we detected longer anaphase time. By contrast, most of the survival cells depleted of Pds5B defectively segregated their chromosomes in a shorter period.

Taken together, the surveillance mechanism is usually activated by DNA damage accompanied by Chk1 activation and decreased S-phase progression. The current work revealed that Pds5A and Pds5B display overlapping functions in facilitating Smc3 acetylation and maintaining genomic integrity. While previous reports suggested that DNA damage-induced Plk1 inhibition is Chk1-dependent and that Plk1 activity increases in Chk1-depleted HeLa cells (Smits et al., 2000; van Vugt et al., 2001; Tang Z. et al., 2006), the current work uncovered a previously unknown property of Chk1 in Pds5-depleted cells, particularly in mitotic arrest. Our findings suggest that Pds5A and Pds5B have non-redundant functions in terms of their impact on the surveillance mechanism that leads to the activation of Chk1 and preventing cohesin removal, as the loss of one cannot be compensated by the presence of the other.

## Materials and Methods

### Cell Culture and Cell Synchronization

Cell culture was carried out at 37°C in a humidified incubator containing 5% CO_2_. HeLa cells were cultured in DMEM (Sigma) supplemented with 10% (v/v) fetal bovine serum (FBS) and 1% (v/v) penicillin/streptomycin solution. RPE1 cells were cultured in DMEM/F-12 (Sigma) supplemented with 0.25% (w/v) NaHCO3, 10% (v/v) FBS, and 1% (v/v) penicillin/streptomycin. For G1/S boundary synchronization, cells were treated with 5 μg/ml aphidicolin (Sigma) for 24 h. For M-phase synchronization, cells were treated with 10 μM Taxol (Sigma) or with 20 nM nocodazole (Sigma) for 24 h and the mitotic cells were harvested by mitotic shake-off. For cell cycle time course, cells were released from aphidicolin block by washing three times with 1X PBS, and supplemented with new fresh complete DMEM and incubated at 37°C in a CO_2_ incubator for a series of time points.

### Antibodies

The following antibodies were obtained commercially: Anti-Pds5A, anti-Pds5B, and anti-Wapl (Bethyl laboratories); anti-Chk1, anti-P-Chk1 (Ser317), anti-histone-H2AX, anti-phospho-Histone-H2AX (Ser139), anti-caspase-3, and anti-cleaved caspase-3 (Cell Signaling Technologies); anti-γ-tubulin, anti-α-tubulin, anti-β-actin, HRP-conjugated goat anti-mouse and HRP-conjugated goat anti-rabbit (Sigma); anti-Esco2 (Novus); human anti-centromere (Bioproduct); anti-Mad2B (BD Transduction Laboratories); anti-acetylated-Smc3 and anti-Rad21/Scc1 (MBL); anti-Smc3 and anti-Cyclin B1 (Santa Cruz Biotechnology); anti-Poly (ADP-ribose) polymerase (PARP) (Roche); anti-Lamin A/C (Abcam); mouse anti-BrdU antibody (BD Biosciences). Alexa Fluor 488 goat anti-mouse IgG, Alexa Fluor 488 goat anti-rabbit IgG, Alexa Fluor 594 Rabbit anti-mouse IgG, and Alexa Fluor 594 goat anti-human IgG (Invitrogen).

### Preparation of Cell Lysates for Immunoblotting and Co-immunoprecipitation

Cells were lysed in radio-immunoprecipitation buffer (RIPA) containing 0.01 M Tris-HCl pH 7.0, 0.15 M NaCl, 2 mM EDTA, 0.1 mM sodium orthovanadate, 0.1% (w/v) sodium dodecyl sulfate (SDS), 1% (v/v) NP-40, 0.5% w/v sodium deoxycholate, 50 mM sodium fluoride (NaF), 30 mM sodium pyrophosphate and protease inhibitor mixture (Roche). For soluble and chromatin-associated protein fractions, cells were lysed with the lysis buffer containing 20 mM Tris-HCl pH 7.5, 100 mM NaCl, 5 mM MgCl_2_, 0.2% (v/v) NP-40, 10% (v/v) Glycerol, 1 mM Sodium Fluoride (NaF), 1 mM Sodium Orthovanadate (Na3VO4), 20 mM β-Glycerophosphate, 10 mM β-Mercaptoethanol, 1% (v/v) Triton X-100, and protease inhibitor mixture and then exposed to a series of three freeze-thaw cycles (−80°C to RT). The DNA pellet were washing three times with 1X PBS (4°C) and lysed with the lysis buffer containing 10 mM Tris-HCl pH7.5, 1 mM CaCl_2_, 1.5 mM MgCl_2_, 0.25 M Sucrose, and 0.008 U/μl micrococcal nuclease and incubated at 28°C for 5 min with gentle mixing. All samples were centrifuged at 4°C at 3000 rpm for 10 min. Soluble and chromatin-associated protein fractions were run on a western blot to assess the levels of proteins bound to chromatin. For Co-immunoprecipitation, Soluble and chromatin-associated protein fractions were incubated with the 0.25 mg of pre-equilibrated protein A sepharose beads (Amersham-Pharmacia) containing 1 μg of primary antibody overnight at 4°C on a rotator. The beads were washed three times with ice-cold lysis buffer and resuspended in 40 μl of SDS-PAGE sample buffer and analyzed by western blot with the appropriate antibodies.

### RNAi

HeLa and RPE cells were transfected with 50 nM siRNAs (Dharmacon) using INTERFERin (Polyplus) for 48 h according to the manufacturer’s protocol. The sequence of the siRNAs used in this study was: Pds5A #1 (5′-GAUAAACGGUGGCGAG UAA-3′), #2 (5′-CCAAUAAAGAUGUGCGUCU-3′), #3 (5′-GA ACAGCAUUGACGACAAA-3′), #4 (5′-GAGAGAAAUAGCCC GGAAA-3′); Pds5B #1 (5′-GAAAUAUGCUUUACAGUCA-3′), #2 (5′-UGAUAAAGAUGUUCGCUUA-3′), #3 (5′-GCAUAGU GAUGGAGACUUG-3′), #4 (5′-GGUCAAUGAUCACUUAC UU-3′); control siRNA (5′-UAGCGACUAAACACAUCAA-3′); Lamin A/C siRNA (5′-ACCAGGUGGAGCAGUAUAA-3′).

### Bromodeoxyuridine (BrdU) Incorporation Assay

BrdU incorporation was performed according to the manufacturer’s protocol. Cells growing on coverslips were treated with siRNAs for 48 h before being synchronized at G1/S with 5 μg/ml aphidicolin for 24 h. Cells were released from the aphidicolin block for different time points and labeled with 1 μM BrdU (Calbiochem) for 30 min in the tissue culture incubator before the end of each time points. Alternatively, exponentially growing cells were labeled with BrdU for 30 min after 48 h transfection with siRNA. After fixing in ice-cold (−20°C) methanol for 5 min at −20°C, cells were hydrolysed with 2 M HCl/0.1% (v/v) Tween 20/PBS for 30 min at RT and blocked with 5% (w/v) BSA/0.1% (v/v) Tween 20/PBS for 1 h prior to staining with a mouse anti-BrdU antibody for 1 h. Then cells were washed with PBS three times and incubated with the Alexa Fluor 488-labeled goat anti-mouse secondary antibody for 1 h. Cells were washed with PBS three times and DNA was stained with 0.5 μM Hoechst 33,342 for 5 min at RT. Cells were visualized and analyzed using immunofluorescence microscopy and for every treatment at least 100 cells were counted in randomly selected fields.

### Fluorescence-Activated Cell Sorting

Analysis of the DNA content was performed by flow cytometry (FACScan, Becton Dickinson) as described previously (Deacon et al., 2003). Briefly, cells were fixed with ice-cold (−20°C) 70% (v/v) ethanol and stored at −20°C. Prior to analysis, the cells were centrifuged at 1100 rpm for 5 min at RT and resuspended in 1X PBS and analyzed using the NucleoCounter^®^ NC-3000^TM^ (Chemometec). Data was analyzed using FlowJo (Version 10.0.6).

### Immunoblotting

Immunoblot analysis was performed as described previously (Deacon et al., 2003).

### Comet Assay

Analysis of DNA damage was performed using the enzyme-modified comet assay protocol, as described previously (Carrera et al., 2013). Briefly, control-si and Pds5-si -treated cells were harvested and 170 μl of low melting point agarose (0.6% agarose (w/v) in PBS) was added to each pellet before 80 μl of each mixture was pipetted onto glass slides pre-coated with 1% (w/v) agarose and allowed to set. Slides were placed in ice-cold lysis buffer containing 100 mM disodium EDTA, 2.5 M NaCl, 10 mM Tris-HCl pH 10.0, 1% (v/v) TritonX-100 for 1 h. Slides were washed with ice-cold distilled water and incubated in ice-cold alkali buffer in the dark for 20 min prior to electrophoresis using electrophoresis buffer containing 300 mM NaOH, 1 mM disodium EDTA pH 13 at 30 V, 300 mA for 20 min. Following their 20 min-incubation in neutralizing buffer (0.4 M Tris-HCl pH 7.5), slides were rinsed in double distilled water and dried overnight at 37°C. Each slide was rehydrated with double distilled water for 30 min and covered with 1 ml of 2.5 μg/ml PI solution (50 μl of 1 mg/ml PI and 20 ml ddH2O) for 20 min at RT in the dark. Slides were washed with double distilled water and oven-dried at 37 °C. Visualization and comet scoring were performed using a fluorescent microscope fitted with the Komet 5.0 imaging system.

### Immunofluorescence Microscopy

Cells were grown on 6-well plates containing sterile glass coverslips. Alternatively, the mitotic cells were harvested by shake-off and were attached to 1 mg/ml poly-L-lysine-coated coverslips. The cells were fixed either with ice-cold 100% methanol for 30 min at −20°C or with 3.7% (v/v) formaldehyde at room temperature for 20 min, followed by permeabilization with 0.1% (v/v) Triton X-100 for 20 min. For some experiments, cells were pre-extracted as previously described (Gandhi et al., 2006). After blocking with 5% (w/v) BSA, the cells were stained with the appropriate primary and secondary antibodies, which were labeled with either Alexa Fluor 488 or 954 fluorescent dyes. Cell nuclei were stained with Hoechst 33342 dye (Sigma). The labeled cells were examined using a Nikon inverted fluorescence microscope (Nikon Eclipse TE 300, Tokyo, Japan) with either an × 100 (NA 1.4) or an × 60 objective (NA 1.4). Images were captured using a Hamamatsu ORCA-R2 digital camera, using Volocity software (Improvision).

### Live-Cell Imaging

HeLa cells stably expressing mCherry-histone H2B and alpha-tubulin-EGFP were grown on 35-mm glass-bottom plates (Matek) in the complete DMEM at 37°C. Following siRNA treatment for 48 h, the medium was replaced with Opti-MEM GlutaMAX containing 10% v/v FBS, and the cells were incubated at 37°C for 1 h before imaging using a Leica SP5 LSCM equipped with a Leica DMI 6000B inverted microscope, which was equipped with a heated stage and a Perspex chamber to maintain an atmosphere of 5% CO_2_. Images were acquired using an ORCA ER CCD camera (Hamamatsu, Japan) and an HCX Plan Apo x63 oil immersion objective (NA 1.4). A z-stack (30–40 sections at 0.3–1 μm) was collected every 8 min throughout 24 h. Images were analyzed, filtered, and processed using Image-J software (Image-J 1.34 s).

### Statistical Analysis

All statistical analyses were typically obtained from a minimum of three independent experiments using Graphpad Prism software (version 6.0). Significant differences were determined using a two-tailed Student’s *t*-test, one-way ANOVA, or two-way ANOVA, as shown in the figure legends, and are indicated with one asterisk (^∗^*P* < 0.05), double asterisks (^∗∗^*P* < 0.01), three asterisks (^∗∗∗^*P* < 0.001), or four asterisks (^****^P < 0.0001).

## Data Availability Statement

All datasets presented in this study are included in the article/Supplementary Material.

## Author Contributions

NA-J and RP designed the experiments. NA-J, LM, and AA performed the research. MA contributed to the new reagents and analytic tools. NA-J and LM analyzed the data. NA-J wrote the manuscript. RP supervised and managed the project. All authors contributed to the article and approved the submitted version.

## Conflict of Interest

The authors declare that the research was conducted in the absence of any commercial or financial relationships that could be construed as a potential conflict of interest.

## Supplementary Material

FIGURE S1 Determination of in frontiers site. the optimal Pds5A and Pds5B SMARTpool siRNAs concentration and duration. **(A,B)** HeLa cells were transfected with the indicated control, Pds5A, or Pds5B SMARTpool siRNAs concentrations. After 48 h, total protein extracts were prepared and subjected to immunoblotting analysis with the antibodies against the indicated proteins. **(C)** Lamin A/C-si (50 nM) were used to test the effectiveness of the transfection procedure. **(D,E)** HeLa cells were transfected with 50 nM of either the control or SMARTpool siRNAs specific for Pds5A **(D)** or Pds5B **(E)**. Total protein extracts were prepared at various time points (6, 12, 24, 30, 36, 48 h) and were analyzed by immunoblotting using the indicated antibodies. **(F)** Immunoblotting analysis Hela cell total lysates with antibodies against the indicated proteins following depletion of Pds5A, Pds5B or both.

MOVIE S1 Control-si-treated HeLa cells undergoing mitosis. Time from prophase to anaphase was 40 min. Scale bar: 10 μm.

MOVIE S2 Pds5A-si-treated cells showing delayed mitosis (time from prophase to anaphase was 200 min) but normal cytokinesis. Scale bar: 10 μm.

MOVIE S3 Pds5B-si-treated cells showing delayed mitosis (136 min from prophase to anaphase). Scale bar: 10 μm.

MOVIE S4 Pds5A-si-treated HeLa cells showing prolonged metaphase arrest (approximately 392 min from prophase) before undergoing cell death. Scale bar: 10 μm.

MOVIE S5 Pds5A-si-treated cells showing prolonged metaphase arrest (approximately 120 min from prophase) before undergoing cytokinesis, with one daughter cell showing the formation of a multi-nucleus and the other daughter cell undergoing cell death. Scale bar: 10 μm.

MOVIE S6 Pds5B-si-treated cells showing a slight mitotic arrest (approximately 288 min from prophase to anaphase) followed by defective cytokinesis and multi-nucleus formation in one daughter cell. Scale bar: 10 μm.

MOVIE S7 Pds5B-si-treated cells failed to align at the metaphase plate and led to a mitotic catastrophe. Scale bar: 10 μm.
