# Supplementary figures and images for: Pds5A and Pds5B Display Non-redundant Functions in Mitosis and Their Loss Triggers Chk1 Activation
Source: Front Cell Dev Biol. 2020 Jul 14;8:531. doi: 10.3389/fcell.2020.00531 (PMC7372117; doi:10.3389/fcell.2020.00531)

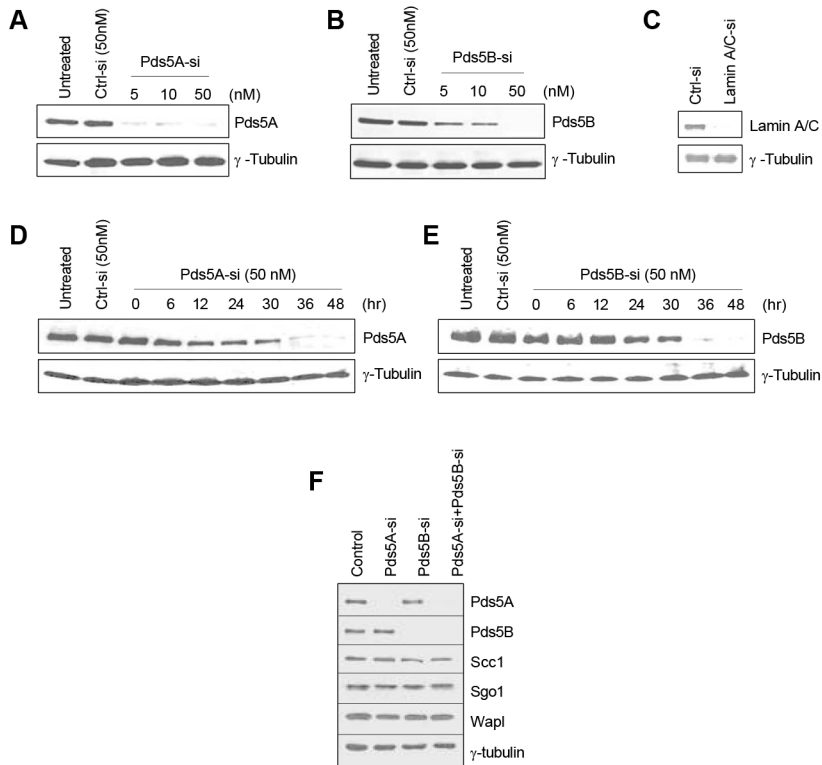

**Figure S1**

Supplement: FIGURE S1 — Determination of in frontiers site. the optimal Pds5A and Pds5B SMARTpool siRNAs concentration and duration. (A,B) HeLa cells were transfected with the indicated control, Pds5A, or Pds5B SMARTpool siRNAs concentrations. After 48 h, total protein extracts were prepared and subjected to immunoblotting analysis with the antibodies against the indicated proteins. (C) Lamin A/C-si (50 nM) were used to test the effectiveness of the transfection procedure. (D,E) HeLa cells were transfected with 50 nM of either the control or SMARTpool siRNAs specific for Pds5A (D) or Pds5B (E). Total protein extracts were prepared at various time points (6, 12, 24, 30, 36, 48 h) and were analyzed by immunoblotting using the indicated antibodies. (F) Immunoblotting analysis Hela cell total lysates with antibodies against the indicated proteins following depletion of Pds5A, Pds5B or both. [file Image_1.pdf]
